# Cochlear implant and large vestibular aqueduct syndrome in children

**DOI:** 10.1016/S1808-8694(15)31098-3

**Published:** 2015-10-19

**Authors:** Trissia Maria Farah Vassoler, Gilberto da Fontoura Rey Bergonse, Silvio Meira, Maria Cecília Bevilacqua, Orozimbo Alves Costa Filho

**Affiliations:** 1 MD. 3rd year ent resident physician; 2 MD. 2nd year ent resident physician; 3 M.D. Full member of the Brazilian College of Radiologists; Radiologist - Imagem Diagnósticos Médicos, Hospital Beneficência Portuguesa de Bauru and Centro de Pesquisas Audiológicas do Hospital de Reabilitação de Anomalias Craniofaciais/USP - Campus Bauru; 4 USP Full Professor - Campus Bauru, Coordinator - interdisciplinary team for the Cochlear Implant Program - Hospital de Reabilitação de Anomalias Craniofaciais da USP - Bauru; 5 USP Full Professor - Campus Bauru, Coordinator - Audiologic Research Center - CPA - Hospital de Reabilitação de Anomalias Craniofaciais da USP - Bauru. Hospital de Reabilitação de Anomalias Craniofaciais (HRAC) - Centro de Pesquisas Audiológicas (CPA) - Universidade de São Paulo (USP) - Bauru/SP

**Keywords:** vestibular aqueduct, cochlear implant, hearing loss

## Abstract

Children with LVAS can develop a severe sensorineural hearing loss early in childhood, but they can be rehabilitated with hearing aids to continue their regular studies and to have a normal life. The problem is that they can deteriorate their hearing capacity, and at this point a cochlear implant can be used to preserve their hearing skills and vocalization.

**Aim:**

to evaluate the hearing skills of 3 children with LVAS referred to cochlear implants.

**Material:**

retrospective study based on medical charts' review.

**Results:**

Speech recognition in open field: patient 1, 80%; patient 2, 87.5%; patient 3, 4 %.

**Conclusion:**

Children with LVAS are considered good candidates for Cochlear implant surgery by the most important centers of the world because most of them can develop good speech recognition, providing them a good social life.

## INTRODUCTION

The vestibular aqueduct is a bony canal within the temporal bone, which goes from the medial wall of the inner ear vestibule all the way to the posterior surface of the petrous bone pyramid. The endolymphatic duct crosses the vestibular aqueduct and ends in the endolymphatic sac. During embryogenesis, the vestibular aqueduct starts as a long and narrow vestibular diverticulum. A development defect before the diverticulum starts to narrow, on the fifth week of gestation, results in an enlarged vestibular aqueduct.^1^

In 1978, Valvassori and Clemis identified 50 cases of enlarged vestibular aqueduct in a retrospective study of 3,700 patients who were submitted to temporal bone CT scan in order to study inner ear structures. A vestibular aqueduct is considered enlarged when its antero-posterior diameter is equal to 1.5mm. These authors were the first to use the definition of Enlarged Vestibular Aqueduct Syndrome (EVAS). Since this time, many studies were carried out in order to better characterize the syndrome and found that 59% to 94% of the cases were bilateral; 60% to 66% of the patients are female, and sensorineural hearing loss is progressive in 46% to 65% of the patients.^1^, ^2^, ^3^, ^4^, ^5^, ^6^^,^^13^^,^^15^

The enlarged vestibular aqueduct may happen as an isolated anomaly or in association with other inner ear malformations. The most common is the enlargement of the horizontal semi-circular canal, 60% to 66%, and cochlear hypoplasia, 28%^7^.

The pathophysiology of this sensorineural hearing loss caused by an enlargement of the vestibular aqueduct is still unknown. Two things may happen, the first suggests a rupture of the labyrinthine membrane or perilymphatic fistula, resulting in a direct transmission of the cerebro-spinal fluid pressure to the middle cochlear turn through the endolymphatic duct and enlarged vestibular aqueduct, and the second suggests a hyperosmolar fluid reflux to the cochlea coming from the endolymphatic sac. More recent studies have mapped the hearing loss associated with the enlarged vestibular aqueduct in the region of chromosome 7q31, however, its clinical meaning still requires more investigation.^1^^,^^12^^,^^15^

Children with EVAS may have moderate to severe hearing deficiencies during their early stages of childhood, however their residual hearing allows them to develop oral language with conventional hearing aids and may be completely integrated to regular school conditions. Nonetheless, these children have a worsening in their hearing skills with time and cochlear implants are being offered as an option to keep their hearing and oral communication skills in proper levels^4^.

This study reports the experience of treating 3 patients with enlarged aqueduct syndrome with cochlear implants.

## MATERIALS AND METHODS

This study was analyzed and approved by the Ethics in Research with Human Beings Committee of the HRAC/USP under protocol # 158/2007-SVAPEPE-CEP.

We carried out a retrospective study to identify all the patients using cochlear implants in our population and were pre-operatively diagnosed with enlarged vestibular aqueducts. So far, 504 cochlear implant surgeries have been performed and only 3 (0.6%) of these patients were diagnosed with enlarged vestibular aqueduct through CT scan and MRI, a much lower number when compared to what has been found in other large centers.

**Figure 1:**
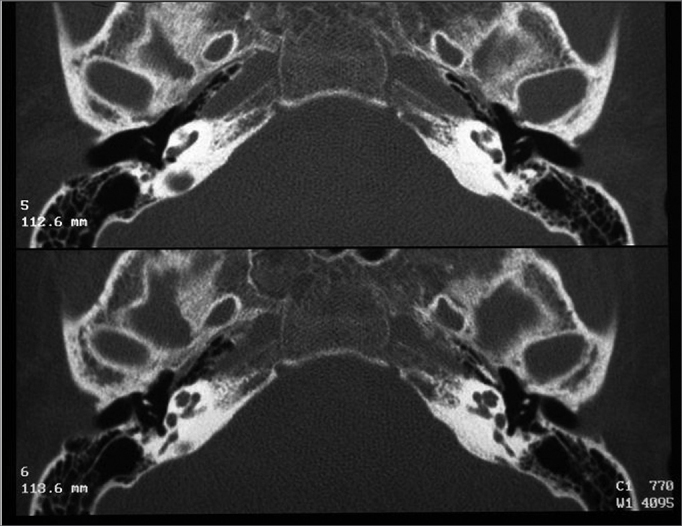
Normal bilateral vestibular aqueduct - Temporal bone CT scan - axial view.

Radiology exams were carried out under sedation with 20% chloral hydrate. CT scans were carried out in a spiral Elscint Twin device, with 0.5mm axial sections, with later axial plane reconstruction (Figure 1).

**Figure 2:**
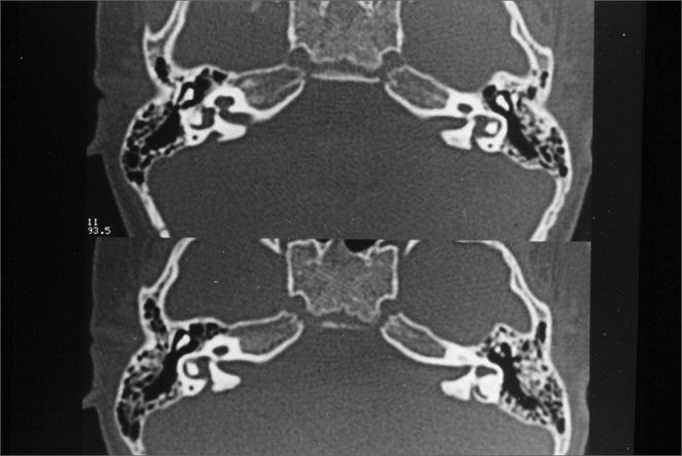
Bilaterally enlarged vestibular aqueduct - temporal bone CT scan - axial view.

The vestibular aqueduct was considered enlarged when its antero-posterior diameter was equal to or greater than 1.5mm (Figure 2).

MRI exams were carried out in Phillips devices with 1.0 Tesla magnetic fields, following the protocol for patients eligible to receive the cochlear implant:

FLAIR sequence for the encephalon.

**Figure 3:**
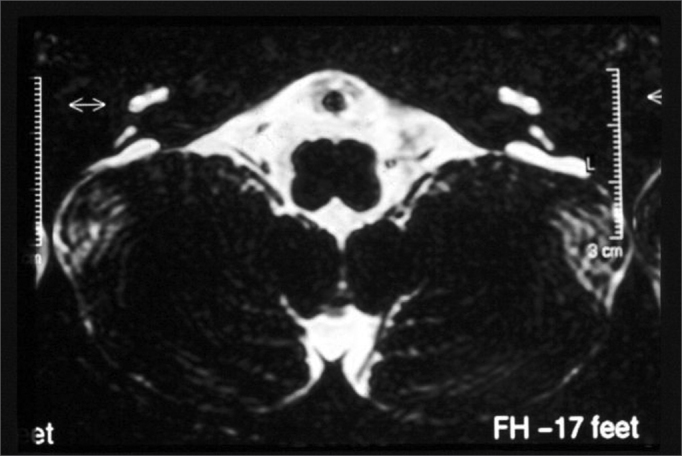
Bilaterally enlarged vestibular aqueduct - temporal bone MRI - axial view in T1 weighed slices.

Turbo SpinEcho (TSE) sequence, axial plane, T1 weighed images of the posterior fossa (Figure 3)

**Figure 4:**
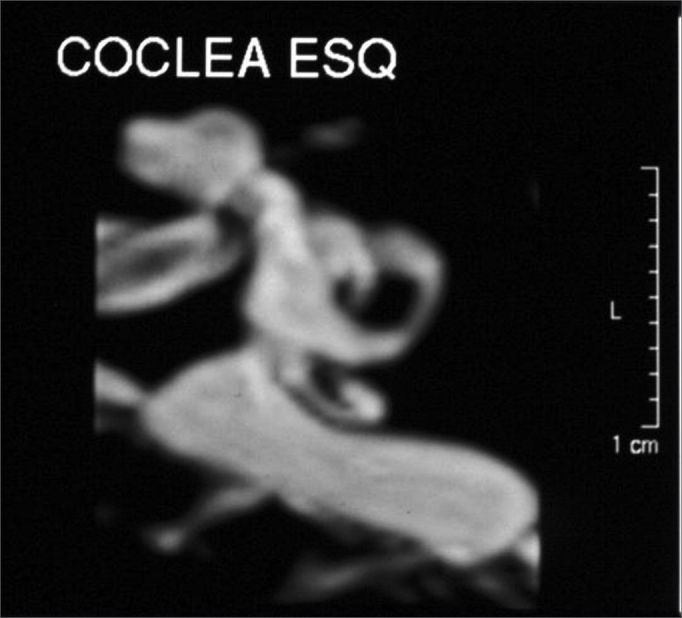
Bilaterally enlarged vestibular aqueduct - temporal bone MRI - axial view - T2 weighed submillimeter slice.

**Figure 5:**
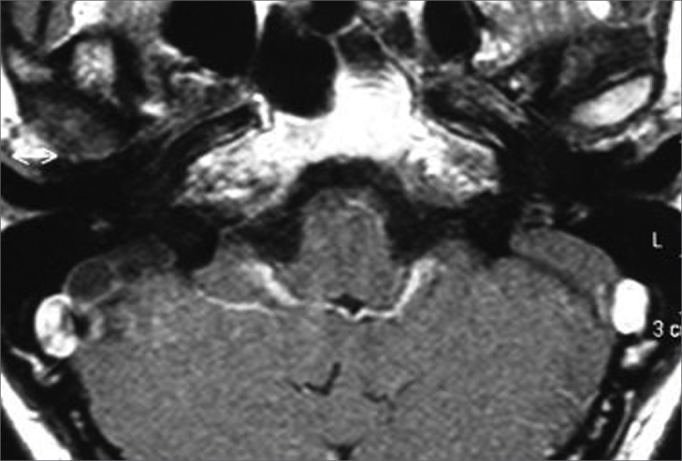
Bilaterally enlarged vestibular aqueduct - temporal bone MRI - axial view - T2 weighed submillimeter slice.

TSE sequence, axial plane, T2 weighed images (Figure 4, Figure 5).

MIP sequence, with submillimeter cross-sections (0.6mm) in the axial and sagittal planes, T2 weighed images, to evaluate the membranous labyrinth and the VII and VIII cranial nerves.

**Figure 6:**
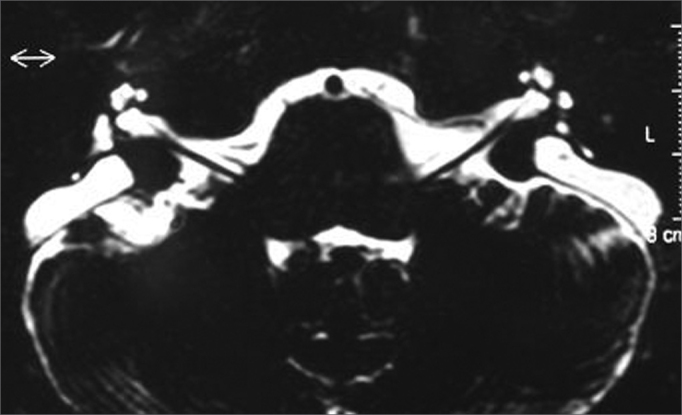
Left side enlarged vestibular aqueduct - Temporal bone MRI, 3D reconstruction.

MIP 3D reconstruction (Figure 6).

## RESULTS

The cochlear implant was done through a transmastoid approach, with posterior tympanotomy and a 2.0mm cochleostomy made in a right angle with the stapes tendon. We had no gusher during cochleostomy, however there was some pulsating oozing of perilymphatic/cerebro-spinal fluid. In all cases the active electrodes were inserted without difficulties, we achieved complete insertion, and the cochlea was sealed with fragments of temporal muscle fascia.

Patient number 1 was male, pre-lingual, and was implanted at 6 years and 5 months of age on the left side.

He received a Nucleus cochlear implant, model CI24RST (Cochlear Corp. Englewood, CO). He had an enlarged vestibular aqueduct and hyperplasic cochlea in the image exam.

Patient 02 was female, post-lingual, and was implanted at the age of 5 years and 9 months, on the right side. She received a Nucleus, CI24RST (Cochlear Corp. Englewood, CO) implant, and did not have other malformations seen in her image exams.

Patient 03 was also a female, pre-lingual, and was implanted at 6 years and 1 month of age, and she was reimplanted three years later, because of a failure in the internal device. She was implanted and reimplanted on the right side and received a MedEl, model C40+ short (MedEl, Innsbruck, Austria) cochlear implant both times, and she did not have other malformations visible in her image exams.

All patients used conventional hearing aids for at least 6 months after the implant and had bilateral enlarged vestibular aqueduct.

Audiometric evaluation for each patient included pure tone recordings without the hearing aid, 6 months of hearing aid use and 1 year after the implant (Table 1).

**Table 1:** Evaluation of pure tone averages of 500, 1000 and 2000 Hz without hearing aid, with hearing aid and with CI.

Patient	1	2	3
Pure tone averages without hearing aid	93.3	106.6	103.3
Pure tone averages with hearing aid	56.6	88.3	83.3
Pure tone averages with CI	26.6	26.6	40.8

Tests from Delgado (8), GASP (9) and Ling's sounds (10) were used to assess speech perception.

Patient # 1 had 80% word recognition, in open settings, patient # 2 had 87.5% in open settings and patient # 3 had word recognition of 4% in open settings, Ling's sound detection was equal to 100%, with phoneme /a/ discrimination equal to 50%, phoneme /i/ equal to 0% and phoneme /u/ equal to 0% in a closed setting.

## DISCUSSION

The Enlarged Vestibular Aqueduct Syndrome (EVAS) represented a treatment challenge for physicians who treated these patients, because of the lack of a protocol able to efficiently prevent the progression of hearing loss in them. Conservative measures such as education to avoid head injuries and barotrauma or pressure fluctuations and treatment with steroids in cases of sudden hearing loss have yielded some success. Endolymphatic sac surgeries were carried out in patients with progressive congenital impairment, however were not considered efficient. Endolymphatic sac obliteration was also described by Wilson et al. as a means to control the progressive hearing loss; however, its results could not be repeated in other studies^1^^,^^15^.

Some few studies have considered the use of a cochlear implant as an option for the patient with EVAS, if bearers of severe-profound or profound sensorineural hearing loss. Harker et al. reported 5 pediatric patients with EVAS, who were implanted and presented excellent results in speech detection. He did not find gusher in any of the surgical procedures. Bent et al. reported a case of 10 patients with EVAS, who received cochlear implant. They noticed a small perilymph pulsating oozing caused by cochleostomy in 5 patients, which were easily controlled with the use of temporal muscle fascia. Seven of the eight children who had already been implanted some time before improved in word recognition in an open setting. Myamoto et al. implanted 23 patients with EVAS, 9 children and 14 adults. He reported gusher in 5 of these patients, however there were no difficulties in inserting electrodes in these patients and the cochleostomy was sealed with a fragment of temporal muscle fascia. This study also found benefits for patients with EVAS^1^.

These results, as well as our experience, consider the cochlear implant surgery to be safe in patients with enlarged vestibular aqueduct and a complete insertion of electrodes is doable. Despite the oozing of perilymph during cochleostomy being somewhat common, it is easily controlled by sealing it with a fragment of temporal muscle fascia. Searching for the association of other malformations with the enlarged vestibular aqueduct and the diameter of the vestibular aqueduct were not relevant in predicting gusher during surgery.^6^

In regards of developing speech skills, there is a marked difference between the many types of inner ear malformations. Unanimously, all cochlear implant centers that have performed surgery in children with inner ear malformations report that those with cochlear dysplasia, enlarged vestibular aqueduct and dilated vestibule, in other words, Mondini's malformation and, especially, those with EVAS are the ones that have the greatest gains in their development with cochlear implants in terms of word recognition and speech^6^^,^^14^. We must also stress that the results are even better in those patients whose hearing loss was installed in the post-lingual phase. As it can be seen with our patients, the post-lingual children had a faster speech perception development when compared to those that had an earlier installed hearing loss. Currently, patients 1 and 2 are evolving very well, and in speech recognition tests present good results in the open setting and most of them can use the telephone in their daily lives. Patient 03 did not do well and other clinical syndromes are being investigated.

Based on the audiometric evaluation of these patients and their speech perception, we can see that these are children with great potential to develop speech perception in an open setting, phonemes and phrases, and this allows them to go to regular schools and have a normal social development. When we notice a low development, we must bear in mind that the enlarged vestibular aqueduct is present in other syndromes such as the Pendred and the Otobrachiorenal syndromes^13^, which impair their neurological development and prevent the children to develop their skills to their maximum.

The major doubt still lies in defining the ideal time for surgery, because hearing loss can behave in different ways such as progressive, sudden or fluctuating, the assessment of these patients require a thorough follow up^11^. The Children's Hospital Implant Center in Sydney, Australia^4^, considers a patient with enlarged vestibular aqueduct eligible for the cochlear implant when the hearing impairment is evident, even using the best hearing aids available, if the periods of inadequate hearing start to impair the child's school performance or if the patient has more than 3 episodes of hearing deterioration in 1 year^4^. We believe the parameters necessary to assess whether or not a patient is eligible for receiving a cochlear implant in cases of enlarged vestibular aqueduct depend on a careful evaluation from a multidisciplinary team, thus, we do not establish specific criteria for this statement.

## CONCLUSION

Patients with enlarged vestibular aqueduct are considered eligible for receiving cochlear implants by the major cochlear implant centers in the world, because most of them develop good audiometric and speech recognition performances, and this provides them better social participation.

Nonetheless, we stress that it is important, besides image studies, to investigate the presence of other syndromes that may follow that of the enlarged vestibular aqueduct.

## References

[1] Miyamoto RT, Bichey BG, Wynne MK, Kirk KI. (2002). Cochlear implantation with large vestibular aqueduct syndrome. Laryngoscope.

[2] Temple RH, Ramsden RT, Axon PR, Saeed SR. (1999). The large vestibular aqueduct syndrome: the role of cochlear implantation in its management. Clin Otolaryngol Allied Sci.

[3] Harker LA, Vanderheiden S, Veazey D, Gentile N, McCleary E. (1999). Multichannel cochlear implantation in children with large vestibular aqueduct syndrome. Ann Otol Rhinol Laryngol Suppl.

[4] Au G, Gibson W. (1999). Cochlear implantation in children with large vestibular aqueduct syndrome. Am J Otol.

[5] Bent JP, Chute P, Parisier SC. (1999). Cochlear implantation in children with large vestibular aqueducts. Laryngoscope.

[6] Buchman CA, Copeland BJ, Yu KK, Brown CJ, Carrasco VN, Pillsbury HC (2004). Cochlear implantation in children with congenital inner ear malformation. Laryngoscope.

[7] Belenky WM, Madgy DN, Leider JS, Becker CJ, Hotaling AJ. (1993). The enlarged vestibular aqueduct syndrome (EVA syndrome). Ear Nose Throat J.

[11] Nakashima T, Ueda H, Furuhashi A, Sato E, Asahi K, Naganawa S (2000). Airbone gap and resonant frequency in large vestibular aqueduct syndrome. Am J Otol.

[12] Lai CC, Shiao AS. (2004). Chronological changes of hearing in pediatric patients with large vestibular aqueduct syndrome. Laryngoscope.

[13] Madden C, Halsted M, Benton C, Greinwald J, Choo D. (2003). Enlarged vestibular aqueduct syndrome in the pediatric population. Otol Neurotol.

[14] Bichey BG, Hoversland JM, Wynne MK, Myamoto RT. (2002). Changes in quality of life and the costutility associated with cochlear implantation in patients with large vestibular aqueduct syndrome. Otol Neurotol.

[15] Bento RF, Lessa MM, Castilho AM, Sanchez TG, Gebrim EMS, Brito Neto RV (2001). Síndrome do aqueduto vestibular alargado: relato de 3 casos e revisão de literatura. Arq Int Otorrinolaringol.

